# Construction on training course and training quality evaluation index system of chronic disease medication therapy management service (MTMs) in China: A Delphi study

**DOI:** 10.1371/journal.pone.0318446

**Published:** 2025-01-30

**Authors:** Wenting Cai, Yao Yao, Wenpu Lei, Huixin Li, Simin Yan, Qiuhui Wu, Jian Wang, Weihong Ge, Jinping Zhang

**Affiliations:** 1 Department of Pharmacy, Nanjing Drum Tower Hospital, School of Basic Medicine and Clinical Pharmacy, China Pharmaceutical University, Nanjing, Jiangsu, China; 2 Department of Pharmacy, Nanjing Drum Tower Hospital, Affiliated Hospital of Medical School, Nanjing University, Nanjing, Jiangsu, China; 3 Department of Pharmacy, Inner Mongolia People’s Hospital, Hohhot, Inner Mongolia, China; 4 Jiangsu Province Hospital of Chinese Medicine, Affiliated Hospital of Nanjing University of Chinese Medicine, Nanjing, Jiangsu, China; Parul University Parul Institute of Technology, INDIA

## Abstract

**Objective:**

This study aims to construct a training course and quality evaluation index system for chronic disease Medication Therapy Management service (MTMs) that is suitable for China’s national conditions. It seeks to provide tools and a scientific foundation for assessing the quality of MTMs training.

**Methods:**

Drawing from domestic and international literature and combining with the practice of chronic disease medication management by Chinese pharmacists, a preliminary framework for the evaluation index system was established. The Delphi method was used to solicit expert opinions, evaluate and improve the evaluation index system. Indicator weights were determined by using the Analytic Hierarchy Process (AHP).

**Results:**

Both rounds of expert inquiry achieved a positive degree of 100% (18/18 experts). The authority coefficients (Cr) were 0.90 and 0.91 respectively. The Kendall coordination coefficients (Kendall’s W) of the second and third-level indicators for the first round of inquiry were 0.230 and 0.189, while those for the second round were 0.326 and 0.213. Finally, an MTMs training course and training quality evaluation index system was structured, comprising 3 first-level indicators, 11 second-level indicators, and 39 third-level indicators.

**Conclusion:**

The evaluation index system constructed in this study is scientifically robust and rational, offering a foundation to standardize MTMs training practices effectively.

## Introduction

Driven by factors such as population aging, urbanization, and changes in lifestyle, the global prevalence of chronic diseases has increased over time [1]. This trend has led to rising rates of disability and mortality worldwide [2]. According to data from the World Health Organization (WHO), cardiovascular diseases remain the leading cause of death globally, responsible for approximately 17.9 million deaths annually, which represents 31% of all global deaths [3]. The number of individuals with diabetes has surpassed 463 million worldwide, with projections indicating that this figure will reach 798 million by 2045 [4]. Chronic respiratory diseases, such as chronic obstructive pulmonary disease (COPD), rank as the third leading cause of death globally, accounting for around 3.2 million deaths each year [5]. In recent years, the rapid aging of the population in China has led to a swift increase in the incidence of chronic diseases, now affecting over 260 million individuals. The rising morbidity and mortality rates underscore the global challenge of managing chronic diseases long-term. Chronic diseases are characterized by high prevalence rates, extended durations of pharmacotherapy, and complex medication regimens [6]. Research indicated that more than 50% of patients with chronic conditions encounter medication-related issues, primarily low adherence, resulting in poor health outcomes and increased healthcare costs. A major factor contributing to low adherence is the lack of proper medication guidance [7, 8]. Pharmacists play an essential role in ensuring safe and rational clinical medication use. Under pharmacists’ guidance, therapy can be more frequently evaluated and individualized treatment plans can be standardized, thereby enhancing the recognition of clinical pharmacy professionals [9, 10]. Consequently, there is significant national emphasis on the role of pharmacists, ensuring the full implementation of their rights and responsibilities.

Currently, pharmaceutical services in China are undergoing a period of transition, with pharmacists transitioning from traditional dispensing to providing comprehensive pharmaceutical services [11]. The traditional pharmaceutical education system and in-service training methods in China exhibit significant differences compared to those in other countries, which have profound implications for pharmacists’ professional practice and service quality. Chinese pharmaceutical education has historically emphasized chemical knowledge and theoretical learning, often neglecting drug therapy management and clinical reasoning training, resulting in a lack of problem-solving skills in complex clinical scenarios [12]. In contrast to Western countries, China has limited in-service training opportunities, which restricts pharmacists’ professional development and the acquisition of updated knowledge [13]. Furthermore, the service model for Chinese pharmacists primarily focuses on drug dispensing and basic consultation, whereas the Western model of medication therapy management offers more comprehensive services, highlighting deficiencies in the education and training systems. The predominance of theoretical education has led to a disconnect between pharmacy education and practical needs, making it challenging for pharmacists to effectively apply their knowledge in clinical practice [12].

Introducing advanced international management approaches to enhance medication oversight for patients with chronic diseases, MTMs serves as a notable model. Originating in the United States during the 1990s, MTMs is defined as a distinctive service designed to optimize pharmacotherapy regimens through comprehensive collection and analysis of patient medical histories, pharmacological monitoring, and related data [6, 14]. The primary objectives of MTMs include the prevention of adverse drug events, enhancement of medication adherence, and promotion of rational drug use via patient education [15]. Since its inception, MTMs has evolved from focusing on acute medication education to addressing the management of medications and associated costs for chronic disease treatment [16]. Studies have demonstrated that MTMs-trained pharmacists who provide specialized services such as medication education and counseling, significantly reduce hospital readmission rates and healthcare costs. It also mitigate medication-related issues, improve patient adherence to prescribed therapies, and prevent medication errors [17–20]. The high demands for practical skills and extensive clinical interactions inherent in the MTMs may not fully align with the existing training background and practical experience of Chinese pharmacists [21]. Consequently, adjustments tailored to China’s specific educational and training frameworks are necessary to better support pharmacists in their role in medication therapy management.

While countries abroad have developed corresponding MTMs training programs and quality evaluation systems, yielding significant results, there remains an absence of a comprehensive MTMs system specifically tailored for chronic diseases. In recent years, an increasing number of pharmacists in China have started to focus on MTMs. However, research related to chronic disease-specific MTMs systems is still lacking. Therefore, under these circumstances, it is imperative to draw on international experience and practice, while considering China’s unique context, to explore the development of a chronic disease MTMs training course and training quality evaluation index system. The objective of this study is to address the research gap in this field. Systematic training content and quality assessment criteria are essential for ensuring consistency and effectiveness in training, thereby enhancing the treatment outcomes and quality of life for chronic disease patients. Clearly defined training standards and quality metrics can assist policymakers in developing more targeted policies and measures, further advancing the standardization and specialization of chronic disease management. Learning from international experience can deepen international cooperation and improve global management standards. The scientific MTMs training course and evaluation system will ensure that pharmacists provide high-quality drug treatment services, improve patient health outcomes, and reduce complications and mortality.

## Methods

### Design

The Delphi method is a qualitative research method used to obtain consensus through expert opinions on real-world problems. In the Delphi method, survey design research with well-structured problems is employed, allowing researchers to obtain accurate and reliable data through multiple rounds of inquiries [22]. The Delphi method features flexible operability and anonymity, with communication between researchers and experts conducted electronically, making it an ideal approach for achieving group consensus. The opinions of experts are not influenced by authority, ensuring the integrity of their input. This study utilized the Delphi method, conducting two rounds of expert consultation to evaluate and discuss preliminary evidence-based indicators from the literature, ultimately achieving consensus among the experts and establishing inquiry indicators. The three-dimensional (Structure-Process-Outcome) quality structure model serves as the theoretical basis for constructing quality evaluation indicators, elucidating the impact of structure and process indicators on outcomes [23]. Based on a comprehensive literature search and analysis, and guided by the "three-dimensional quality structure model", a preliminary evaluation index system for MTMs training courses and training quality was developed. The weights of indicators at all levels were determined using the AHP.

### Establishing a research group

Our research group consisted of 9 experienced pharmaceutical experts, clinical pharmacists, and clinical pharmacy students. Among them, there were 2 individuals holding a senior professional title, 3 with an associate senior professional title, 1 with an intermediate professional title, and 3 clinical pharmacy graduate students. The main tasks of our research group included literature review, collaborative development of questionnaire indicators for inquiry, selection of inquiry experts, analysis of expert feedback, and statistical analysis of data. Our research group’s role encompassed discussions and analysis of evidence-based indicators, as well as the creation of a preliminary draft of key indicators. Additionally, we established an expert inquiry form and determined the members of the Delphi expert inquiry group. Efforts were made to identify experts with substantial and profound understanding of chronic disease medication training, in order to gather their opinions and perspectives on the index system developed in this study.

### Design initial inquiry questionnaire

**Fig 1:**
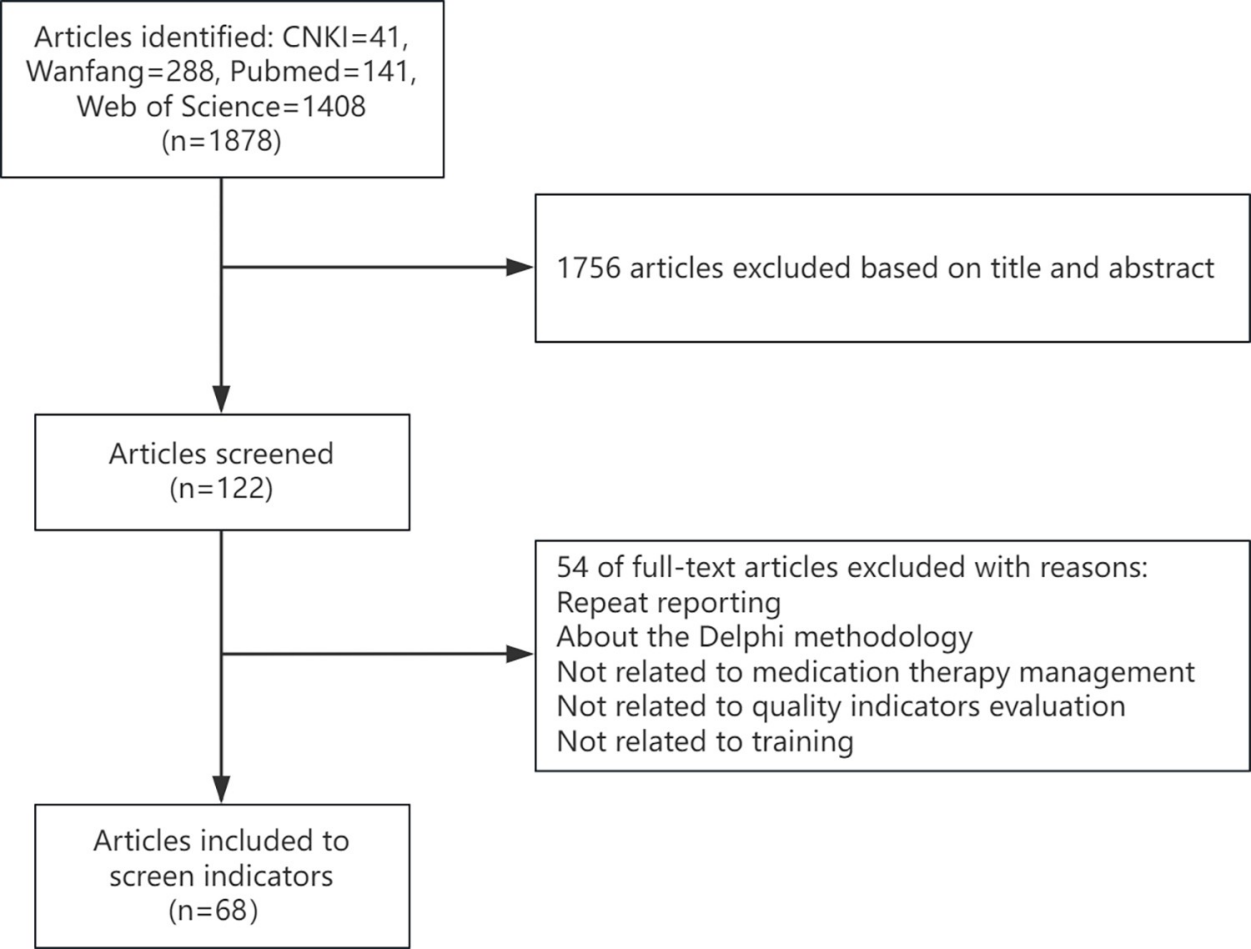
The flow chart of literature screening.

We utilized databases such as CNKI, Wanfang, Pubmed and Web of Science from the inception of each database to October 2021 for literature retrieval. The main search terms were as follows: "Delphi" and "Chronic Disease”. We selected these broad terms because using more restrictive terms might not capture all relevant articles of interest. In the aforementioned databases, we conducted a comprehensive search for relevant papers, filtering based on titles, abstracts, keywords, subject terms and references. Two members thoroughly reviewed the full texts of the included studies to extract and summarize the data. The literature search process was illustrated in **Fig 1**. We screened evaluation indicators and preliminarily developed expert inquiry questionnaires. Ultimately, the selection of indicators was refined through expert interviews. The inquiry questionnaire consists of five parts. (1) Questionnaire description: This section included research background, research purpose, and the questionnaire return time. (2) Research methods and table filling instructions: This part provided an explanation of the research methods and instructions on how to complete the tables. (3) Basic information table of experts: This included information such as age, position, educational background, professional title, years of employment, research field, and teaching experience. (4) Questionnaire subject: This section included description and explanation of indicators, an importance rating table, and expert feedback columns. (5) Scoring table: This portion scored the experts’ familiarity with the indicators and the influence of the judgment basis on experts.

### Expert inclusion criteria

According to the Delphi method, the appropriate number of experts is 15–50 [24]. The inclusion criteria for experts in this study were: (1) At least 6 years of experience in MTMs-related fields and extensive experience in pharmaceutical teaching or training; (2) Holding a bachelor’s degree or higher; (3) Holding an intermediate or higher professional title; (4) Strong interest and enthusiasm for the study and willingness to serve as an expert; (5) Willingness to provide valuable feedback and continue participating until the end of inquiry. Experts provided opinions and suggestions on the questionnaire content through two rounds of questionnaire inquiry, and scored the importance of each indicator. They expressed their opinions independently and remained anonymity to one another. The research group determined the final quality evaluation system based on expert suggestions, combined with the categories and descriptions of indicators. Therefore, expert opinions and suggestions determined the authority of the final quality evaluation system.

### Delphi expert questionnaire inquiry

To ascertain inquiry responses, the questionnaires were disseminated to experts via email and collected within a week. The evaluation of each indicator was based on the expert positivity degree, expert authority level, and expert opinion coordination level. The expert authority coefficient (Cr) was determined by the scores of the expert familiarity (Cs) and the degree of influence of the judgment basis on experts (Ca). The Cr was calculated based on Cs and Ca, using the formula Cr = (Cs+Ca)/2. The experts’ familiarity with the research content is categorized into five levels: very familiar, relatively familiar, generally familiar, not very familiar, and very unfamiliar, corresponding to values of 1.0, 0.8, 0.6, 0.4, and 0.2, respectively. The influence of the judgment basis of on experts’ assessment of indicators is assigned as shown in **Table 1**. The degree of opinion coordination was determined by examining the coefficient of variation (CV) and the Kendall’s W. The initial expert inquiry questionnaire consisted of 3 first-level indicators, 13 second-level indicators, and 46 third-level indicators. The Likert 5-level scoring method was used to score the importance of each indicator [25]. A score of 5 is very important, 4 is relatively important, 3 is generally important, 2 is relatively unimportant, and 1 is very unimportant. Experts were encouraged to provide additional insights, including the ability to supplement, delete, or integrate any indicators, as well as offering alternative viewpoints.

**Table 1:** Quantitative table of the influence of the judgment basis.

Judgment basis	The degree of influence of the judgment basis on experts		
	Large	Medium	Small
Practical experience	0.5	0.4	0.3
Theoretical analysis	0.3	0.2	0.1
Reference to domestic and foreign literatures	0.1	0.1	0.1
Intuitive judgment	0.1	0.1	0.1

### Questionnaire indicators screening criteria

Upon completion of the inquiry, the experts’ scoring data was subjected to statistical analysis, and the "boundary value method" was employed to screen the tertiary indicators. The analysis included calculating the mean (Mj), full score rate (Kj), and CV for each indicators based on their importance scores. The threshold for selecting indicators is determined as follows: for the Mj and the Kj, the threshold is defined as "threshold = mean—standard deviation," and indicators with scores exceeding this threshold will be retained. For the CV, the threshold is defined as "threshold = mean + standard deviation," and indicators with scores below this threshold will be selected. Indicators failing to meet all three criteria were eliminated. For those not meeting one or two criteria, further discussion with experts was conducted to reach a decision [26]. This iterative process formed the basis for the subsequent round of the expert inquiry questionnaire.

### Statistical methods

We used Excel to input data and calculate the Mj, Kj and CV for each indicator’s importance score. The Kendall’s W was calculated by SPSS software. The experts positive degree was expressed by the effective response rate of the questionnaires. The Cr was represented by the arithmetic mean of Cs and Ca. The degree of expert opinion coordination was evaluated by the CV and the Kendall’s W, with the latter being tested using the chi-square test (*P*<0.05 indicating statistical significance). The indicators weight was calculated by using Yaahp software.

### Analytic Hierarchy Process

To construct a judgment matrix, we assigned a mean value to the importance of each indicator as rated by the experts. Using a nine-point scale (refer to **2**), we compared the importance of the indicators in pairs to determine the Saaty scale [27], thereby converting qualitative assessments into quantitative ratings. Based on this judgment matrix, we employed Yaahp software to automatically calculate the weights of each indicator by the AHP, and we analyzed the indicators accordingly. The consistency of the judgment matrix is verified using the consistency ratio (CR), where a CR < 0.1 indicates that the judgment matrix possesses acceptable consistency.

**Table 2:** A nine-point scale for pairwise comparison.

Intensity of importance	Definition	Description
1	Equal importance	Both items contribute equally
3	Slight importance	One item is slightly more important than the other
5	Moderate importance	One item is moderately more important than the other
7	Strong importance	One item is strongly more important than the other
9	Extreme importance	One item is extremely more important than the other
2, 4, 6, 8	Intermediate values between two adjacent judgements	Compromised judgement is needed
Reciprocals	If item A is assigned the certain number when compared with item B, the item B is assigned the reciprocal value of the certain number when compared with item A	

## Results

### Basic information of experts

This study involved two rounds of expert inquiry, during which consensus was achieved on expert opinions, leading to the conclusion of the inquiry. In the first round, 18 experts were invited, with an average age of (42.78 ± 6.48) years and an average work experience of (18.11 ± 7.98) years. They comprised 1 professor, 2 associate professors, 3 chief physicians, 3 chief pharmacists, 7 associate chief pharmacists and 2 pharmacist-in-charge. All the experts held academic qualifications at the undergraduate level or above, with more than half possessing a master’s degree. They were affiliated with tertiary hospitals or pharmaceutical universities across the country and had extensive teaching and practical experience in chronic disease management and MTMs training. The basic information of the experts was shown in **Table 3**. Furthermore, these experts also participated in the second round of inquiry.

**Table 3:** Basic information of inquiry experts.

	Project	Number	Constituent ratio
Age	30~39	7	38.89%
	40~49	9	50.00%
	50~59	2	11.11%
Sex	Male	8	44.44%
	Female	10	55.56%
Region	Jiangsu	5	27.78%
	Shandong	3	16.67%
	Anhui	2	11.11%
	Chongqing	2	11.11%
	Guangdong	2	11.11%
	Shanghai	2	11.11%
	Xinjiang	2	11.11%
Education	Undergraduate	3	16.66%
	Master	10	55.56%
	Doctor	5	27.78%
Work experience	6~10 years	2	11.11%
	11~15 years	7	38.89%
	16~20 years	4	22.22%
	Over 20 years	5	27.78%
Professional title	Pharmacist-in-charge	2	11.11%
	Associate chief pharmacist	7	38.89%
	Chief pharmacist	3	16.67%
	Chief physician	3	16.67%
	Associate professor	2	11.11%
	Professor	1	5.55%
Working field	Clinical pharmacy	10	55.56%
	Pharmacy administration	4	22.22%
	Chronic disease management	4	22.22%

### Expert positivity degree

According to the Delphi research method, a recovery rate of 50% is considered the minimum ratio for analysis and reporting, with 60% being deemed good and 70% very good [28]. In this study, 18 expert questionnaires were distributed in the first round, and all 18 valid questionnaires were collected. The positivity coefficient of experts was 100%, and 50% of experts proposed modification suggestions. In the second round, 18 expert questionnaires were again distributed, and all 18 valid responses were collected, maintaining a positivity coefficient of 100% among the experts. This indicated that both rounds demonstrated high enthusiasm from the participating experts.

### Expert authority coefficient

The Cr value fluctuates between 0 and 1, and a value greater than 0.7 is considered acceptable. When Cr≥0.8, the authority level is considered high, and the opinions given are more reliable [29]. In this study, the Cr for two rounds of expert inquiry were 0.90 and 0.91, respectively, indicating a very high level of expert authority, as shown in **Table 4**.

**Table 4:** Authority coefficients for two rounds of expert inquiry.

Inquiry round	Number	Cs	Ca	Cr
Round one	18	0.88	0.92	0.90
Round two	18	0.89	0.92	0.91

Cs: Expert familiarity; Ca: The degree of influence of the judgment basis on experts; Cr: Expert authority coefficient.

### Degree of coordination of expert opinions

The degree of opinion coordination refers to the extent of agreement among experts on various indicators, expressed by the CV and Kendall’s W. A smaller CV indicates higher consistency among experts, while Kendall’s W ranges from 0 to 1, with higher values denoting greater expert coordination and more reliable results [30]. In the two rounds of expert inquiry, the experts scored the importance of the first, second, and third-level indicators. In the first round of inquiry, the CV of each level indicator fluctuated between 0.10 and 0.16, and the Kendall’s W for the second and third-level indicators were 0.230 and 0.189, respectively. The second round of the CV ranged from 0.05 to 0.16, with Kendall’s W of 0.326 and 0.213 for the second and third-level indicators, respectively. The results from the second round of inquiry indicated an increase in Kendall’s W for both the second and third-level indicators. The *χ*^*2*^-test results demonstrated statistical significant (*P*<0.05), as shown in **Table 5**, suggesting a high level of coordination among the experts’ opinions. This indicated that the expert opinions were largely unanimous.

**Table 5:** Coordination coefficients for two rounds of expert inquiry.

Inquiry round	Project	Second level indicators	Third level indicators
Round one	Kendall’s W	0.230	0.189
	*χ* ^*2*^	46.607	156.202
	*P*	0.000	0.000
Round two	Kendall’s W	0.326	0.213
	*χ* ^*2*^	58.717	149.208
	*P*	0.000	0.000

### Indicators screening

Based on the evaluation results of the "boundary value method" and expert opinions, indicators screening were conducted following a discussion. The boundary values for two rounds of expert inquiry were shown in **Table 6**.

**Table 6:** The table of boundary values for two rounds of expert inquiry.

Indicators	Project	First round of expert inquiry		Second round of expert inquiry	
		Mean+/-standard deviation	Boundary values	Mean+/-standard deviation	Boundary values
First level indicators	Mj	4.72–0.06	4.66	4.96–0.06	4.90
	Kj	0.72–0.06	0.66	0.96–0.06	0.90
	CV	0.10+0.01	0.11	0.02+0.04	0.06
Second level indicators	Mj	4.66–0.23	4.43	4.72–0.28	4.44
	Kj	0.69–0.19	0.50	0.74–0.23	0.51
	CV	0.11+0.04	0.15	0.09+0.05	0.14
Third level indicators	Mj	4.60–0.20	4.40	4.70–0.25	4.45
	Kj	0.63–0.16	0.47	0.74–0.18	0.56
	CV	0.11+0.03	0.14	0.10+0.05	0.15

Mj: Mean; Kj: Full score rate; CV: Coefficient of variation.

In the first round, no adjustments were made to the first-level indicators. Several second-level and third-level indicators were deleted or retained based on boundary value criteria and expert feedback (**Table 7**). Indicators that need to be revised or added according to expert opinions were as follows:

"1.1.4 Establish WeChat Groups": Updated to "1.1.4 Establish Various Communication Platforms" to include multiple platforms like WeChat, QQ, Youxun, and DingTalk for improved communication.

"2.5.4 Case Discussion": Added to emphasize practical experience and enhance clinical practice and teaching effectiveness. Experts indicated that the ultimate goal of training was to better serve clinical practice. Case discussion simulates clinical practice, promoting communication skills, pharmaceutical service attitudes, and accumulating clinical experience, thereby significantly improving teaching effectiveness.

“3.2.1 Professional competence”, “3.2.2 communication skills”, “3.2.3 Professional quality”, “3.2.4 Patient Management”: Revised to “3.2.1 Professional knowledge level”, “3.2.2 Practical ability”, “3.2.3 Communication skills”, “3.2.4 Professional quality” respectively. These revisions better reflected the focus on cultivating specific abilities and implementing a progressive evaluation system for student development.

"3.3.1 Student satisfaction", "3.3.2 Patient satisfaction", "3.4.1 Scientific research output", "3.4.2 Awards": These indicators were removed due to the elimination of their preceding level indicators and because they were deemed difficult to measure and of limited significance.

In the second round, the expert opinions have basically reached a consensus, with only supplementary adjustments made to the description and explanation of the third-level indicators. All indicators met the three conditions of the "boundary value method". After two rounds of inquiry and adjustment, the final index system consisted of 3 first-level indicators, 11 second-level indicators, and 39 third-level indicators. The changes in the number of questionnaire indicators were shown in Table 8.

**Table 7:** Indicators that need to be were deleted or retained based on boundary value criteria and expert feedback in the first round.

Indicators	Mj	Kj	CV	Explanation
2.2 Student Qualifications	4.433	**0.500**	**0.177**	Retained despite not meeting two criteria due to its importance in assessing students’ backgrounds and ensuring effective training design.
3.1 Academic Record	**4.389**	**0.444**	0.138	Retained despite failing two criteria for its role in evaluating students’ post-training mastery and progress.
3.3 Satisfaction	**4.333**	**0.444**	**0.158**	Deleted because three criteria were not met regarding the two indicators. Experts agreed that demanding substantial output from students in a short period was unrealistic.
3.4 Output Situation	**4.389**	**0.500**	**0.159**	
1.1.3 Group Management of Students	**4.333**	**0.389**	0.137	Retained with two conditions met, as it promoted peer support and collective progress.
1.4.3 Teaching Emergency Plan	4.444	**0.444**	0.115	Retained despite meeting only one condition, reflecting its importance for ensuring teaching continuity during emergencies.
2.2.2 Operating Post	**4.389**	**0.444**	0.138	Deleted due to failure to meet required conditions regarding the two indicators, with a shift in focus to broader student participation.
2.2.3 English Level	**4.000**	**0.278**	**0.192**	
2.4.1 Online Teaching	**4.278**	**0.444**	**0.176**	Deleted for not meeting three conditions, prioritizing offline training with online options as secondary.
2.5.1 Introduction to Class Start	**4.389**	**0.444**	0.138	Deleted as redundant, with the obligation that students were already aware of course introductions.

The bold part indicates items that did not satisfy the threshold method.

**Table 8:** Description of changes in questionnaire indicators.

Indicators		Initial questionnaire	First round of expert inquiry	Second round of expert inquiry
First level indicators	Outcome	4	4	4
	Structure	5	5	5
	Process	4	2	2
Second level indicators		13	11	11
Third level indicators		46	39	39

### Analytic Hierarchy Process for calculating indicator weights

In this study, all CR values of the judgment matrix were less than 0.1, indicating the successful completion of the consistency test. The final MTMs training course and training quality evaluation index system were shown in **Table 9**, along with the corresponding indicator weight outcomes.

**Table 9:** The evaluation index system and indicator weight results.

Indicators	Weight		Combined Weight		Combined Weight
1.1.1 Complete roles and reasonable organizational structure	0.2622	1.1 Organization structure	0.2529	1 Structure quality	0.3294
1.1.2 Clear job responsibilities of each teaching management personnel	0.2591				
1.1.3 Group management of students	0.2378				
1.1.4 Establish various communication platforms	0.2409				
1.2.1 Personnel management system	0.2530	1.2 Working system	0.2500		
1.2.2 Course management system	0.2530				
1.2.3 Platform management system	0.2470				
1.2.4 Assessment management system	0.2470				
1.3.1 Generally feasible training plan	0.3437	1.3 Training program	0.2558		
1.3.2 Reasonable requirements for teaching objectives	0.3242				
1.3.3 Reasonable training cycle	0.3320				
1.4.1 Stable operation of teaching platform	0.3413	1.4 Facility support	0.2413		
1.4.2 Organizational support	0.3374				
1.4.3 Teaching emergency plan	0.3213				
2.1.1 Love teaching	0.2019	2.1 Teacher selection	0.2006	2 Process quality	0.3333
2.1.2 Teaching qualifications	0.1972				
2.1.3 Professional title requirements	0.1878				
2.1.4 Working Experience	0.2089				
2.1.5 Teaching Experience	0.2042				
2.2.1 Education and Professional Title	1.0000	2.2 Student qualifications	0.1883		
2.3.1 Reasonable course content design	0.2589	2.3 Course Design	0.2077		
2.3.2 Appropriate frequency of new course launch	0.2441				
2.3.3 Diversified teaching methods	0.2500				
2.3.4 Value the dissemination of tacit knowledge	0.2470				
2.4.1 Group discussion	0.5031	2.4 Training mode	0.1935		
2.4.2 Online Q&A	0.4969				
2.5.1 Baseline survey	0.1906	2.5 Learning process	0.2100		
2.5.2 Course learning	0.1964				
2.5.3 Mid-term tutoring	0.1964				
2.5.4 Case discussion	0.2107				
2.5.5 Graduation assessment	0.2059				
3.1.1 Learning completion status	0.2438	3.1 Academic record	0.4730	3 Outcome quality	0.3373
3.1.2 After class assessment score	0.2480				
3.1.3 Case analysis score	0.2561				
3.1.4 Graduation assessment score	0.2522				
3.2.1 Professional knowledge level	0.2449	3.2 Ability promotion	0.5270		
3.2.2 Practical ability	0.2479				
3.2.3 Communication skills	0.2507				
3.2.4 Professional literacy	0.2565				

## Discussion

The questionnaires were distributed via email, which saved time for the experts and increased their enthusiasm for participation. The positive coefficients of the two rounds of expert inquiry were both 100%, with the Cr of 0.90 and 0.91, respectively. Therefore, the response and authority levels of the experts in this study were high, making the inquiry results reliable. Regarding the coordination of expert opinions, the CV for the third-level indicators were all lower than 0.25, and the Kendall’s W for the second and third-level indicators were 0.230, 0.189, and 0.326, 0.213, respectively. After performing a χ2-test, the results were statistically significant (P<0.05). Consequently, the experts strong agreement with the indicators, and their evaluation opinions were consistent, indicating broad recognition of the study’s results by the experts.

### The scientificity and rationality of the evaluation index system

This study mainly used the Delphi method and AHP to construct an MTMs training course and training quality evaluation index system based on the SPO concept. The system included 3 first-level indicators, 11 second-level indicators, and 39 third-level indicators, providing a comprehensive tool for evaluating MTMs training quality. The results of this study are desirable, reasonable and scientific. Firstly, the formulation of the index system in this study was grounded in the SPO quality structure model, which is well-established for developing evaluation indicators for teaching and training quality [31]. Secondly, by fully searching domestic and foreign literature and integrating it with the practical work experience of Chinese pharmacists, the system was refined through two rounds of Delphi expert questionnaire surveys and extensive discussions, ensuring high credibility. Thirdly, the representativeness and professionalism of the selected experts enhance the authority of the research results. The selection of experts is the key to the success of the Delphi method. For this study, experts engaged in clinical pharmacy, pharmaceutical management, and chronic disease management related majors were chosen. These experts included both those proficient in theoretical research and those with extensive practical experience. They possess significant representativeness in their professional fields, and their qualifications in terms of education, age, professional title, specialties, and years of experience meet the requirements of the Delphi method [23, 32].

### Explanation of index system weights

The weight results showed that among the first-level indicators, the proportion of Structure quality was 0.3294, Process quality was 0.3333, and Outcome quality was 0.3373. In 1969, American scholar Donabedian proposed a theoretical model of SPO which opened up new perspectives for quality evaluation across three dimensions [33]. “Structure” pertains to the training environment, which refers to the man and material resources required to complete the training. “Process” refers to the direct or indirect training content received by students, essentially how structural attributes are applied in training practice. "Outcome" evaluates the success of training implementation. The structure, process, and outcome are interrelated and exhibit a linear relationship: a robust structure significantly increases the likelihood of a favorable process, which in turn positively impacts the outcome [34]. Donabedian noted that Although both structure and process are important, the end outcome is often decisive in the assessment of training quality [33]. This is consistent with the findings from this study, where outcome quality was given the most weight. Based on this relationship, scoring the importance of the Structure, Process and Outcome quality of MTMs training, including secondary indicators at each stage, can be better inform the construction of a quality evaluation index system and improve overall training quality [34].

Training program (0.2558) accounted for the highest proportion of Structure quality. The design of the training program should thoroughly address the educational background and needs of pharmacists in Chinese hospitals, ensuring practicality and a precise reflection of the entire training process. Clear objectives aligned with the learners’ educational and professional requirements can notably enhance the effectiveness of the training. This aligns with Shelton’s view that training programs should be tailored to the actual needs of learners to better achieve training goals [35]. Organization structure (0.2529) highlighted its crucial role in the training process. Clear role distribution and responsibility management facilitate the smooth execution of the training. Establishing various platforms for learning, communication, and discussion, and grouping students according to their educational background and professional field, can significantly improve training efficiency. This organizational design is consistent with Parkinson and Lowe’s recommendation that professional development requires clear organizational support and resources [36].

In terms of Process quality, Learning process (0.2100) was identified as the most crucial aspect. Firstly, a comprehensive assessment of students’ foundational knowledge before the training begins aids in tailoring learning plans to their level. This approach aligns with Brauer’s experiential learning theory, which emphasizes enhancing learning outcomes through practice and feedback [37]. During the learning process, chapter-end quizzes and expert responses to student queries allow for timely adjustments in teaching strategies, improving the specificity and effectiveness of learning. Notably, Case discussions (0.2107), as a significant form of practical application, effectively translate theoretical knowledge into practical skills through large group discussions and expert evaluations. This method is supported by Spinella et al., who argued that case discussions significantly enhance learners’ ability to apply knowledge [38]. Course Design (0.2077) also played a vital role in Process quality. The course content must be closely aligned with educational objectives while considering the learning background of pharmacists in Chinese hospitals. The curriculum, which includes drug therapy, evidence-based medicine, and case discussions, facilitates the integration of theory and practice. This approach is consistent with Diggele and Burgess, who found that diverse teaching methods enhance learning outcomes [39]. Furthermore, various teaching methods in course design, such as Lecture-Based Learning (LBL), Case-Based Learning (CBL), and Problem-Based Learning (PBL), accommodate different learning styles and improve teaching effectiveness [40]. Teacher selection (0.2006) highlighted the importance of professional qualifications and practical experience. Teachers are required to hold a master’s degree or higher and have relevant experience in MTMs. This aligns with Kozhevnikova’s research, which indicates that teacher expertise is crucial for educational quality [41]. Additionally, practical experience is considered a key component of effective teaching [42].

In the context of Outcome quality, Ability promotion (0.5270) was emphasized as a critical part. Ability promotion includes four key areas: Professional knowledge level (0.2449), Practical ability (0.2479), Communication skills (0.2507), and Professional literacy (0.2565). A study suggested that medical training should particularly focus on cultivating these four aspects [43]. Whitehurst highlighted that the level of knowledge among healthcare professionals directly affects the quality of clinical decision-making and patient outcomes [44]. Training programs must ensure that students acquire up-to-date medical knowledge and apply it effectively in practice. Payakachat et al. found that practical experience helps healthcare professionals handle complex clinical situations more effectively, thus enhancing their overall capabilities [42]. Therefore, training should emphasize the development of practical skills through simulation and hands-on practice. Ruiz-Moral discovered a significant correlation between healthcare professionals’ communication skills and patient outcomes and satisfaction [45]. Consequently, training should focus on improving communication skills, including effective interaction with patients and team members. Research indicated that the level of professionalism affects both healthcare professionals’ behavior and patient trust [46]. Therefore, training should incorporate education on professional ethics and behavior standards to enhance overall competence. For comprehensive assessment, it is recommended to use self-assessment, peer evaluations among team members, and evaluations by training experts. This multi-faceted approach can improve assessment accuracy and reliability, providing a more complete understanding [47].

Weight analysis can provide decision support, help decision makers to formulate strategies more objectively and scientifically, and explain the rationality and credibility of research results. At the same time, it provides guidance and reference for follow-up research.

### The advantages of the evaluation index system

This index system possesses unique value in several ways. (1) Systematic and Standardized: The system is based on expert consultation using the Delphi method, resulting in a comprehensive and standardized training curriculum. This systematic approach not only facilitates its adoption across China but also has significant international potential, ensuring consistency and high standards in training processes and outcomes. (2) Strong Relevance: The system is designed specifically to address the practical needs of pharmacists involved in chronic disease management, tailoring course content to clinical practice requirements and effectively enhancing pharmacists’ professional capabilities in this area. This focus addresses the generalization issues prevalent in existing systems. (3) Clear Evaluation Mechanism: This study has developed explicit quality evaluation indicators for training, providing a scientific basis for assessing the effectiveness of training programs. This systematic evaluation mechanism ensures continuous improvement in training quality and offers actionable references for future enhancements.

Compared to existing indicator evaluation systems, this system offers distinct advantages. (1) Relevance of Course Content: Existing training systems often emphasize general pharmaceutical education, whereas this system focuses on the specific needs of chronic disease management pharmacists, aligning course content with practical applications. Existing systems frequently overlook the special requirements of chronic disease management, a gap this system fills. (2) Systematic and Integrated Approach: Current training systems may suffer from fragmented and non-systematic content. In contrast, this study integrates diverse expert opinions using the Delphi method, resulting in a comprehensive, systematic training curriculum that addresses the integration deficiencies of existing systems. (3) Scalability and Adaptability: While existing systems have achieved some success in certain regions, their scalability is limited by resources and geographic constraints. The course and management model designed in this study offer significant scalability and, through the development of skilled trainers, can be implemented nationwide, enhancing training quality and effectiveness and advancing the standardization of chronic disease management pharmacist education. These unique designs and improvements not only significantly enhance pharmacists’ capabilities in chronic disease management but also provide valuable insights for pharmaceutical education in China and beyond, fostering comprehensive development in pharmaceutical education and chronic disease management.

### Limitations

This study still has some limitations. (1) After limited time screening, we only selected 18 experts for the Delphi method inquiry, with only three more experts than the minimum number required by the Delphi method. Moreover, these experts mainly come from hospitals in China that attach great importance to and have developed well in pharmacist education and training. The education level and work experience of the experts also vary, so there may be biases in the research results. We will select more experts from different regions and hospitals in future research, and try to include more expert opinions including small samples. Through statistical analysis, we will achieve more comprehensive consistency to improve our evaluation index system. (2) Due to the use of email to send questionnaires to experts, we were unable to provide explanations on specific issues, and experts may have a deviation in their understanding of the indicators. A reliable and effective questionnaire needs to pass the test of reliability and validity. We will distribute the questionnaire evaluation form to clinical pharmacists and experts from representative hospitals across the country, in order to obtain more research data on chronic disease MTMs training and optimize the design of our index system. (3) Due to the lack of empirical application testing, our next step is to conduct practical teaching empirical research on this index system and further analyze its applicability.

## Supporting information

S1 File (DOCX)

S2 File (DOCX)

S3 File (DOCX)
