# Supplementary material for: Construction on training course and training quality evaluation index system of chronic disease medication therapy management service (MTMs) in China: A Delphi study
Source: PLoS One. 2025 Jan 30;20(1):e0318446. doi: 10.1371/journal.pone.0318446 (PMC11781749; doi:10.1371/journal.pone.0318446)
Supplement: S1 File — (DOCX) [file pone.0318446.s001.docx]

**MTMs培训课程与培训质量评价指标体系专家函询问卷（第一轮）**

尊敬的专家：

您好！非常感谢您在百忙之中参加本次函询！

我国已进入人口老龄化社会，老年人慢性病患病率高，多病共患、多药共用，由此导致的不合理用药日益增多。为慢病患者提供药物治疗管理服务，是药师的重要任务，也是药师药学服务能力的体现。但我国药师在校期间接受的是以化学模式为主的药学教育，如何开展适合我国药师学习基础的慢病管理课程培训与教学管理评价、有效提高药师慢病管理能力，是促进药学发展与药师转型的重要工作。本研究拟构建适合我国国情与药师学习基础的慢病管理服务培训课程与培训质量评价指标体系，诚邀您提出宝贵建议！

在前期研究中，课题组已初步构建了包括**结构质量、过程质量、结果质量三**个层级的评价指标体系，含有**3项一级指标、13项二级指标与46项三级指标**。本问卷针对指标的重要性与可行性进行函询，期待您在百忙之中于一周内提出修改意见与建议。请通过微信返回给我或发送至邮箱：[zjp16500@163.com](mailto:zjp16500@163.com)。

衷心感谢您的支持与帮助，祝您工作顺利！

MTMs培训课题组

2023年4月23日

**一、研究方法和填表说明**

本次研究采用德尔菲法进行至少两轮专家函询，请每位专家依据Likert 5 级评分法（从“非常不重要”～“非常重要”为1 ～ 5 分）对各指标重要性进行评分，请您在合适的框内打“√”。若您认为该项指标不需要或者不准确，可在 “专家意见”填写栏中提出意见及建议。**请您在1 周内填写问卷并返回**，我们将对回收问卷进行数据整理和意见汇总，为避免记忆影响的偏倚，我们将在2周后向您发放修订后的函询表，并进行第二轮专家咨询。此问卷为匿名形式整理，且专家之间互不知道哪些专家参加函询。

**二、专家一般情况**

| 您的年龄： |  |
| --- | --- |
| 您的职务： |  |
| 您的学历： |  |
| 您的职称： |  |
| 您的从业年限： |  |
| 您的主要研究领域： |  |
| 您是否有过教学质量评价指标体系建设的经历？ | A是，具体角色有：①组织者；②参与者；③顾问；④其他 _______（可多选）  B否 |

**三、问卷内容（请在相应栏内打“ √ ”，新增指标也在相应栏内打“√”）**

**表1 一级指标咨询表**

| **一级指标** | **重要性** | | | | | **专家意见（修改为）** |
| --- | --- | --- | --- | --- | --- | --- |
|  | **非常不重要**  1分 | **比较不重要**  2分 | **一般**  3分 | **比较重要**  4分 | **非常重要**  5分 |  |
| **1结构质量** |  |  |  |  |  |  |
| **2过程质量** |  |  |  |  |  |  |
| **3结果质量** |  |  |  |  |  |  |
| **需增加的指标** |  | | | | | |
| **需删减、整合或调整的指标，及其他建议** |  | | | | | |

**表2 二级指标咨询表**

| **一级指标** | **二级指标** | **指标说明** | **重要性** | | | | | **专家意见（修改为）** |
| --- | --- | --- | --- | --- | --- | --- | --- | --- |
|  |  |  | **非常不重要**  1分 | **比较不重要**  2分 | **一般**  3分 | **比较重要**  4分 | **非常重要**  5分 |  |
| **1结构质量** | **1.1组织架构** | 教学团队人员分工明确，建立有效交流平台 |  |  |  |  |  |  |
|  | **1.2工作制度** | 合理的人员、课程、平台、考核管理制度 |  |  |  |  |  |  |
|  | **1.3培训方案** | 培训目标明确，培训周期合理 |  |  |  |  |  |  |
|  | **1.4设施保障** | 教学平台稳定，应急预案切实可行 |  |  |  |  |  |  |
| **2过程质量** | **2.1师资遴选** | 具有硕士以上学历、中级以上职称，丰富的工作经验和教学经验 |  |  |  |  |  |  |
|  | **2.2学员资质** | 具有本科以上学历、药师以上职称，大学英语四级以上水平 |  |  |  |  |  |  |
|  | **2.3课程设计** | 内容设计合理，教学手段多样性 |  |  |  |  |  |  |
|  | **2.4培训模式** | 线上授课、分组讨论、在线答疑 |  |  |  |  |  |  |
|  | **2.5学习过程** | 开班启动、基线调查、课程学习与辅导、案例讨论、结业考核 |  |  |  |  |  |  |
| **3结果质量** | **3.1学习成绩** | 包括学习完成情况和课后考核、案例分析、结业考核成绩 |  |  |  |  |  |  |
|  | **3.2能力提升** | 知识水平、实践能力、沟通能力、职业素养都得到提升 |  |  |  |  |  |  |
|  | **3.3满意度** | 学员对培训的满意度、患者对药师的满意度、学员单位对培训效果的满意度不低于80% |  |  |  |  |  |  |
|  | **3.4产出情况** | 药学门诊工作的规范性得到了提高，相关论文、课题、奖项等产出增加 |  |  |  |  |  |  |
| **需增加的指标** |  | | | | | | | |
| **需删减、整合或调整归类的指标，及其他建议** |  | | | | | | | |

**表3 指标描述咨询表**

| **一级指标** | **二级指标** | **三级指标** | **指标说明** | **重要性** | | | | | **专家意见（修改为）** |
| --- | --- | --- | --- | --- | --- | --- | --- | --- | --- |
|  |  |  |  | **非常不重要**  1分 | **比较不重要**  2分 | **一般**  3分 | **比较重要**  4分 | **非常重要**  5分 |  |
| **1结构质量** | **1.1组织架构** | **1.1.1教学团队中，角色齐全，组织架构合理** | 设置教学总负责人、教学执行负责人、授课老师、教学秘书以及培训组长等相关角色 |  |  |  |  |  |  |
|  |  | **1.1.2各教学管理人员岗位职责明确** | 教学管理人员岗位职责明确，覆盖了对教学全过程的管理 |  |  |  |  |  |  |
|  |  | **1.1.3对学员进行分组管理** | 根据学员学历、职称、专业领域进行分组 |  |  |  |  |  |  |
|  |  | **1.1.4建立微信群** | 分别建立全体学员与各组学员微信群，为开展学员之间的学习交流与讨论做好准备 |  |  |  |  |  |  |
|  | **1.2工作制度** | **1.2.1人员管理制度** | 建立完善的人员管理制度，教学团队中，人员职责明确、分工合理 |  |  |  |  |  |  |
|  |  | **1.2.2课程管理制度** | 建立完善的课程管理制度，课程安排、课时分布、课后测验井然有序 |  |  |  |  |  |  |
|  |  | **1.2.3平台管理制度** | 建立教学平台管理制度，专人负责平台运行，有序进行课程发布、后台管理工作 |  |  |  |  |  |  |
|  |  | **1.2.4考核管理制度** | 建立规范的考核管理制度，合理设置成绩权重、结业标准与考核方式 |  |  |  |  |  |  |
|  | **1.3 培训方案** | **1.3.1培训方案总体可行** | 培训方案充分考虑中国医院药师学习基础与慢性病学习需求，切实可行，真实反映培训全过程 |  |  |  |  |  |  |
|  |  | **1.3.2教学目标要求合理** | 教学目标明确，切合学员业务工作需求 |  |  |  |  |  |  |
|  |  | **1.3.3培训周期合理** | 培训周期合理，课程安排兼顾学员工作与业余时间 |  |  |  |  |  |  |
|  | **1.4设施保障** | **1.4.1教学平台运行稳定** | 线上教学平台运行稳定，界面友好，操作方便 |  |  |  |  |  |  |
|  |  | **1.4.2组织支撑** | 药学类教指委作为主办单位，对教学中遇到的各种问题能够积极协调、及时解决，确保教学工作顺利推进 |  |  |  |  |  |  |
|  |  | **1.4.3教学应急预案** | 为确保教学活动的正常进行，对于教学中可能发生的异常情况，建有应急预案 |  |  |  |  |  |  |
| **2过程质量** | **2.1师资遴选** | 2.1.1热爱教学 | 热爱教学，对教学工作具有较高热情 |  |  |  |  |  |  |
|  |  | 2.1.2教学资质 | 获得美国明尼苏达大学 MTMs培训证书或具有与课程教学相匹配的资质条件 |  |  |  |  |  |  |
|  |  | 2.1.3职称条件 | 具备中级及以上职称 |  |  |  |  |  |  |
|  |  | 2.1.4工作经验 | 在慢性病领域任职5年以上，具有丰富的专业知识与药物管理治疗技能 |  |  |  |  |  |  |
|  |  | 2.1.5教学经验 | 在慢性病领域任职5年以上，具有丰富的实践教学经验 |  |  |  |  |  |  |
|  | **2.2 学员资质** | **2.2.1学历与职称** | 具有大学本科及以上学历、药师及以上专业技术职称 |  |  |  |  |  |  |
|  |  | **2.2.2工作岗位** | 在本单位承担慢病患者药学服务相关岗位工作 |  |  |  |  |  |  |
|  |  | **2.2.3英语水平** | 具有大学英语四级及以上水平，可胜任英文文献查阅与读译工作 |  |  |  |  |  |  |
|  | **2.3课程设计** | **2.3.1课程内容设计合理** | 教学内容紧扣教学目标，兼顾中国医院药师的学习基础，课程内容涵盖常见慢性疾病的药物治疗学、循证医学、药物经济学以及药物治疗管理方法学；安排案例讨论教学，帮助学员实现从理论学习向实际运用的跨越 |  |  |  |  |  |  |
|  |  | **2.3.2新课上线频率适当** | 依据学习内容的难易程度，合理安排新课上线频率 |  |  |  |  |  |  |
|  |  | **2.3.3教学方法多样化** | 运用CBL、LBL、PBL等多种教学手段，丰富教学形式，增强教学效果 |  |  |  |  |  |  |
|  |  | **2.3.4重视隐性知识的传播** | 通过积极引导，促进隐性知识的传播，提高教学效果 |  |  |  |  |  |  |
|  | **2.4培训模式** | **2.4.1线上授课** | 将线上平台与手机软件相结合，开展课程教学，充分利用学员的碎片时间 |  |  |  |  |  |  |
|  |  | **2.4.2分组讨论** | 为促进隐性知识的传播，将学员分组，学员在组内开展学习讨论、交流学习心得 |  |  |  |  |  |  |
|  |  | **2.4.3在线答疑** | 对于组内讨论无法解决的问题，安排授课教师在线答疑 |  |  |  |  |  |  |
|  | **2.5学习过程** | **2.5.1开班启动** | 培训正式开始前，向学员介绍教学安排、教学平台使用方法、学习与考核要求，设置每周1-2节新课的上线提醒 |  |  |  |  |  |  |
|  |  | **2.5.2基线调查** | 培训开始前，对学员进行摸底考试，了解学员的学习基础 |  |  |  |  |  |  |
|  |  | **2.5.3课程学习** | 每周上线1-2章节教学课程，学员按照学习计划完成课程学习。每章节学习结束，学员完成课后测验，得分在70分及以上者合格 |  |  |  |  |  |  |
|  |  | **2.5.4中期辅导** | 在培训中期，征集学员在学习中遇到的问题，汇总后分类，组织中期辅导会，邀请相关专家、授课老师答疑 |  |  |  |  |  |  |
|  |  | **2.5.5结业考核** | 全部培训课程结束后，组织结业考核，平时成绩合格者方可参加结业考核 |  |  |  |  |  |  |
| **3结果质量** | **3.1学习成绩** | **3.1.1学习完成情况** | 完成全部线上课程的学习 |  |  |  |  |  |  |
|  |  | **3.1.2课后考核成绩** | 在规定时间内完成课后测验，且均分不低于70分 |  |  |  |  |  |  |
|  |  | **3.1.3案例分析成绩** | 按要求提交案例，按照MTMs的步骤进行案例分析并书写表单，经授课老师评分后成绩合格 |  |  |  |  |  |  |
|  |  | **3.1.4结业考核成绩** | 结业考核成绩合格 |  |  |  |  |  |  |
|  | **3.2能力提升** | 3.2.1**专业能力** | 培训结束时，学员自评其药学专业能力得到了提升 |  |  |  |  |  |  |
|  |  | **3.2.2沟通能力** | 培训结束时，学员自评其沟通能力得到了提升 |  |  |  |  |  |  |
|  |  | **3.2.3职业素养** | 培训结束时，学员自评其职业素养、工作态度得到了提升 |  |  |  |  |  |  |
|  |  | **3.2.4患者管理** | 培训结束时，学员自评其慢病患者用药管理水平得到了提升 |  |  |  |  |  |  |
|  | **3.3满意度** | **3.3.1学员满意度** | 学员的培训满意度不低于80% |  |  |  |  |  |  |
|  |  | **3.3.2患者满意度** | 患者对药师的满意度不低于80% |  |  |  |  |  |  |
|  | **3.4产出情况** | **3.4.1**科研产出 | 通过培训，相关论文、课题等产出增加 |  |  |  |  |  |  |
|  |  | **3.4.2**获奖情况 | 以药物治疗管理为主题，在相关学术活动中奖项 |  |  |  |  |  |  |
| **需增加的指标** |  | | | | | | | | |
| **需删减、整合或调整归类的指标，及其他建议** |  | | | | | | | | |

**四、判断依据与熟悉程度**

**1.根据您对评价指标的熟悉程度，在表4中相应的空格中打“ √ ”。**

**2.对指标进行判断时，通常会不同程度地受到实践经验、理论分析、国内外文献报道和直觉等因素的影响，请您根据这四个方面因素影响您做出判断的程度大小，分别在相应空格中打“ √ ”。**

**表4 专家对填表内容熟悉程度的自我评价**

| **很熟悉** | **较熟悉** | **一般熟悉** | **不太熟悉** | **不熟悉** |
| --- | --- | --- | --- | --- |
|  |  |  |  |  |

**表5 专家对填表内容判断依据的自我评价**

| **判断依据** | **对专家判断的影响程度** | | |
| --- | --- | --- | --- |
|  | **大** | **中** | **小** |
| **实践经验** |  |  |  |
| **理论分析** |  |  |  |
| **参考国内外文献** |  |  |  |
| **直觉判断** |  |  |  |
