# Supplementary material for: Construction on training course and training quality evaluation index system of chronic disease medication therapy management service (MTMs) in China: A Delphi study
Source: PLoS One. 2025 Jan 30;20(1):e0318446. doi: 10.1371/journal.pone.0318446 (PMC11781749; doi:10.1371/journal.pone.0318446)
Supplement: S3 File — (DOCX) [file pone.0318446.s003.docx]

**慢病管理药师培训课程构建专家函询问卷（第一轮）**

**尊敬的专家：**

**您好！非常感谢您在百忙之中参加本次函询！**

**我国已进入人口老龄化社会，老年人慢性病患病率高，多病共患、多药共用，由此导致的不合理用药日益增多。对慢病患者进行药物治疗管理，是药师的重要任务，也是药师药学服务能力的体现。但我国药师在校期间接受的是以化学模式为主的药学教育，如何开展适合我国药师学习基础的慢病管理课程培训与教学管理评价，有效提高药师慢病管理能力，是促进药学发展与药师转型的重要工作。本研究拟通过文献调研、专家函询与实证研究，构建适合我国国情与药师学习基础的慢病管理药师课程培训体系与培训质量评价指标体系。诚邀您为慢病管理药师培训体系与质量评价指标的构建工作提出宝贵建议！**

**一、慢病管理药学服务工作开展情况**

1、您认为下列哪些慢性病发病率高，为临床常见慢性病（以下简称“慢病”）？

A高血压病 B糖尿病 C慢性阻塞性肺疾病

D支气管哮喘 E消化性溃疡 F冠心病 G骨关节炎

H慢性疼痛 I脑卒中 J心力衰竭 K甲状腺功能减退

2、与感冒、发热等疾病的药学服务相比，针对慢病患者的药学服务有何不同？

A药师对患者进行长期随访 B药师须具备药物治疗学知识

C药师须具备较强的沟通能力 D药师须具备药物治疗管理技能

3、如果药师为慢病患者提供药学服务，可在哪些场所提供？

A三级医院门诊 B三级医院住院病房

C二级医院门诊 D二级医院住院病房

E社区卫生服务中心

4、您是否听说过药学服务门诊？

A听说过 B没有听说过

5、在您周围，有无医疗机构开设药学服务门诊？

A有 B不清楚 C没有

5、您认为，药学服务门诊是否适合开展慢病患者管理工作？

A适合 B不适合

6、您周围医疗机构的药学服务门诊，是否开展了慢病患者管理工作？

A已开展 B不清楚 C未开展

7、从患者的角度出发，您认为慢病患者管理应当包含哪些服务内容？

A提供用药指导 B解答用药咨询 C向慢病患者提供长期用药管理

D收集药物不良反应 E其他，具体为：

8、如果您周围的药学服务门诊开展了慢病患者管理工作，您认为他们的工作是否规范？

A 很规范 B一般般 C不规范

9、您心目中规范的慢病患者管理工作，应包含哪些方面？

A为患者建立用药档案 B提供用药指导 C对患者进行随访

D收集患者疗效相关信息 E管理患者合并症的药物治疗信息

F向医生或其他医疗机构转诊 G解答用药问题 H收集不良反应

10、如果药师慢病患者管理工作不够规范，您认为可能的原因有哪些？

A药师专业知识欠缺 B工作繁忙，时间有限 C缺乏培训

D绩效管理不到位 E缺乏对药师工作的监管

11、药师为慢病患者提供药学服务中，较为关键的服务有哪些？

A提供用药指导 B进行药物重整 C进行长期随访

D与患者建立良好的合作 E及时解决患者提出的用药问题

F能够从患者角度解决问题 G及时提醒患者随访时间

12、据您了解，药师为慢病患者提供药学服务时，存在的困难有哪些？

A缺乏收费标准 B培训不到位 C药师间交流不够

D缺乏工作标准 E领导不重视 F医师不了解药师的工作

G患者不了解药师的工作 H药师发现用药问题时，缺乏解决问题的机制 I前来接受服务的患者少 J患者失访率高 K患者依从性差

13、是否有必要开展针对慢病管理药师的课程培训？

A有必要 B没必要

**二、慢病管理药师培训课程**

14、参加慢病管理培训的药师，需要具备哪些条件？

A具备临床药师或药学门诊工作经历 B具有良好的沟通能力

C有耐心 D具有一定的文献查阅与英文资料阅读能力

E乐观开朗，积极向上 F工作努力，热爱药学事业

15、您认为，慢病管理药师应具备哪些知识与技能？

A药物治疗学知识 B文献查阅与筛选能力 C疾病严重程度评估能力 D治疗方案的遴选能力 E个体化给药方案制定能力

F药物相互作用分析 G疗效评估能力 H药学监护能力

G沟通能力

16、慢性病管理药师的培训课程，具体应包含哪些内容？

A常见慢性病的药物治疗学知识 B循证医学知识

C药物经济学知识 D药物治疗管理方法学

E沟通技巧 F案例分析与讨论

17、您认为培训采用哪些形式较为合适？

A采用线上教学平台培训 B线下教学 C线上线下相结合

18、您认为，影响培训效果的因素有哪些？

A学生学习不认真 B教师授课技巧不够 C课程安排不合理

D学生间互动少 E师生间互动少 F教学管理不到位

G学习目标不明确，学习与应用相脱离 H考试流于形式

19、为做好培训工作，需要关注以下哪些问题？

A加强对学习的督查与管理 B设立教学管理组织

C引导学生提问 D加强学生间、师生间互动

E及时鼓励 F树立榜样 G严格考核

**三、慢病管理药师培训考核**

20、您认为应从哪些方面来评价慢病管理药师培训质量？

A结构质量 B过程质量 C结果质量

21、您认为结构质量具体可从哪些方面得以体现？

A开展培训的硬件设施 B软件资源 C师资资源

D管理模式 E培训工作制度 F管理措施 G其他

22、您认为过程质量具体可从哪些方面得以体现？

A学员的作业完成率 B学员对培训课程的满意度 C学员对培训内容的理解和掌握程度 D课后测验得分 E案例分析书写质量评分 F结业答辩成绩

23、您认为结果质量具体可从哪些方面得以体现？

A向慢病患者提供服务后，患者疗效的提高

B治疗费用的下降 C患者满意度的上升

D其他，具体为：
